# Determinants of Mortality in a Combined Cohort of 501 Patients With HIV-Associated Cryptococcal Meningitis: Implications for Improving Outcomes

**DOI:** 10.1093/cid/cit794

**Published:** 2013-12-06

**Authors:** Joseph N. Jarvis, Tihana Bicanic, Angela Loyse, Daniel Namarika, Arthur Jackson, Jesse C. Nussbaum, Nicky Longley, Conrad Muzoora, Jacob Phulusa, Kabanda Taseera, Creto Kanyembe, Douglas Wilson, Mina C. Hosseinipour, Annemarie E. Brouwer, Direk Limmathurotsakul, Nicholas White, Charles van der Horst, Robin Wood, Graeme Meintjes, John Bradley, Shabbar Jaffar, Thomas Harrison

**Affiliations:** 1 Research Centre for Infection and Immunity, Division of Clinical Sciences, St George's University of London, United Kingdom; 2 Desmond Tutu HIV Centre, Institute of Infectious Disease and Molecular Medicine, University of Cape Town, South Africa; 3 Department of Clinical Research, Faculty of Infectious and Tropical Diseases, London School of Hygiene and Tropical Medicine, United Kingdom; 4 Division of Infectious Diseases and HIV Medicine, Department of Medicine, University of Cape Town, South Africa; 5 University of North Carolina Project, Lilongwe, Malawi; 6 Division of Infectious Diseases, University of California, San Francisco; 7 Mbarara University of Science and Technology, Uganda; 8 Edendale Hospital, Pietermaritzburg, South Africa; 9 St Elisabeth Hospital Tilburg and Radboud University Medical Centre Nijmegen, the Netherlands; 10 Mahidol-Oxford Research Unit, Faculty of Tropical Medicine, Mahidol University, Bangkok, Thailand; 11 Centre for Tropical Medicine, Nuffield Department of Clinical Medicine, University of Oxford, United Kingdom; 12 Institute of Infectious Disease and Molecular Medicine, University of Cape Town, South Africa; 13 Department of Medicine, Imperial College London, United Kingdom; 14 Faculty of Epidemiology and Population Health, London School of Hygiene and Tropical Medicine, United Kingdom

**Keywords:** cryptococcal meningitis, *Cryptococcus neoformans*, HIV, antiretroviral therapy, mortality (determinants)

## Abstract

***Background.*** Cryptococcal meningitis (CM) is a leading cause of death in individuals infected with human immunodeficiency virus (HIV). Identifying factors associated with mortality informs strategies to improve outcomes.

***Methods.*** Five hundred one patients with HIV-associated CM were followed prospectively for 10 weeks during trials in Thailand, Uganda, Malawi, and South Africa. South African patients (n = 266) were followed for 1 year. Similar inclusion/exclusion criteria were applied at all sites. Logistic regression identified baseline variables independently associated with mortality.

***Results.*** Mortality was 17% at 2 weeks and 34% at 10 weeks. Altered mental status (odds ratio [OR], 3.1; 95% confidence interval [CI], 1.7–5.9), high cerebrospinal fluid (CSF) fungal burden (OR, 1.4 per log_10_ colony-forming units/mL increase; 95% CI, 1.0–1.8), older age (>50 years; OR, 3.9; 95% CI, 1.4–11.1), high peripheral white blood cell count (>10 × 10^9^ cells/L; OR, 8.7; 95% CI, 2.5–30.2), fluconazole-based induction treatment, and slow clearance of CSF infection were independently associated with 2-week mortality. Low body weight, anemia (hemoglobin <7.5 g/dL), and low CSF opening pressure were independently associated with mortality at 10 weeks in addition to altered mental status, high fungal burden, high peripheral white cell count, and older age.

In those followed for 1 year, overall mortality was 41%. Immune reconstitution inflammatory syndrome occurred in 13% of patients and was associated with 2-week CSF fungal burden (*P* = .007), but not with time to initiation of antiretroviral therapy (ART).

***Conclusions.*** CSF fungal burden, altered mental status, and rate of clearance of infection predict acute mortality in HIV-associated CM. The results suggest that earlier diagnosis, more rapidly fungicidal amphotericin-based regimens, and prompt immune reconstitution with ART are priorities for improving outcomes.

## Abstract (precis)

Cerebrospinal fluid fungal burden, altered mental status, and rate of clearance of infection predict acute mortality in HIV-associated cryptococcal meningitis. The identification of factors associated with mortality informs strategies to improve outcomes.

Human immunodeficiency virus (HIV)–associated cryptococcal meningitis (CM) is the commonest cause of adult meningitis in much of Africa [1–4]. Despite antifungal treatment, acute mortality in the developing world remains between 24% and 43% [5–7], and CM accounts for 10%–20% of all HIV-related deaths in sub-Saharan Africa [8]. The median time to death following hospital admission with CM is 10–13 days [6]. To develop evidence-based interventions, it is essential to determine the key predictors of mortality. Using data from a cohort of 501 patients with CM from Thailand, South Africa, Malawi, and Uganda, we describe the presenting clinical features and outcomes of patients with HIV-associated CM, and report the results of a predictive model used to identify the clinical and microbiological factors at baseline independently associated with mortality. We provide an analysis of factors associated with altered mental status, cerebrospinal fluid (CSF) fungal burden, CSF opening pressure (OP) at presentation, rate of clearance of infection, and immune reconstitution inflammatory syndrome (IRIS).

## METHODS

The cohort comprised patients from 9 trials conducted from 2002 to 2010 at 5 sites (Table 1) in Thailand, South Africa, Malawi, and Uganda. The trials have been reported elsewhere, and represent all trials of HIV-associated CM published (at the time of analysis) using early fungicidal activity (EFA) as the primary outcome [5, 9–16]. A previous analysis of 262 patients explored the correlation between rate of clearance of infection and survival [17]. Combining the data from the constituent trials into a combined cohort was done to obtain the power needed to reliably determine the predictors of mortality in patients with HIV-associated CM. All trials were sponsored by St George's University of London and approved by the St George's Research Ethics Committee and local ethics committees.

**Table 1.** Component Studies Contributing to the Combined Cohort

Author and Type of Study^a^	Site and Year	No. of Subjects	Induction Treatment^b^	ART Available	EFA, log_10_ CFU/mL/d, Mean (SD)
Brouwer et al [9] RCT	Thailand 2002	64	AmB 0.7 mg/kg/d (n = 16)	No	−0.31 (0.18)
			AmB 0.7 mg/kg/d + 5-FC 100 mg/kg/d (n = 16)		−0.54 (0.19)
			AmB 0.7 mg/kg/d + Fluc 400 mg/d (n = 16)		−0.39 (0.15)
			AmB 0.7 mg/kg + 5-FC 100 mg/kg + Fluc 400 mg/d (n = 16) (All for 14 d)		−0.38 (0.13)
Bicanic et al [10] Cohort study	South Africa 2005	54	AmB 1 mg/kg/d for 7 d then Fluc 400 mg/d (n = 49)	Yes	−0.48 (0.28)
			Fluc 400 mg/d for 14 d (n = 5)		−0.02 (0.05)
Bicanic et al [5] RCT	South Africa 2005–2006	64	AmB 0.7 mg/kg/d + 5-FC 100 mg/kg/d (n = 30)	Yes	−0.45 (0.16)
			AmB 1 mg/kg/d + 5-FC 100 mg/kg/d (n = 34) (Both for 14 d)		−0.56 (0.24)
Longley et al [11] Cohort study	Uganda 2005–2007	60	Fluc 800 mg/d (n = 30)	Yes	−0.07 (0.17)
			Fluc 1200 mg/d (n = 30) (Both for 14 d)		−0.18 (0.11)
Nussbaum et al [12] RCT	Malawi 2008	41	Fluc 1200 mg/d (n = 20)	Yes	−0.11 (0.10)
			Fluc 1200 mg/d + 5-FC 100 mg/kg/d (n = 21) (Both for 14 d)		−0.28 (0.17)
Loyse et al [13] RCT	South Africa 2006–2008	80	AmB 1 mg/kg/d + 5-FC 100 mg/kg/d (n = 21)	Yes	−0.41 (0.22)
			AmB 1 mg/kg/d + Fluc 800 mg/d (n = 22)		−0.38 (0.18)
			AmB 1 mg/kg/d + Fluc 1200 mg/d (n = 24)		−0.41 (0.35)
			AmB 1 mg/kg/d + Vori 600 mg/d (n = 13) (All for 14 d)		−0.44 (0.20)
Muzoora et al [14] Cohort study	Uganda 2008–2009	30	AmB 1 mg/kg/d for 5 d + Fluc 1200 mg/d for 14 d (n = 30)	Yes	−0.3 (0.11)
Jackson et al [15] RCT	Malawi 2009–2010	40	AmB 1 mg/kg/d for 7 d + Fluc 1200 mg/d for 14 d (n = 20)	Yes	−0.39 (0.20)
			AmB 1 mg/kg/d for 7 d + Fluc 1200 mg/d and 5-FC 100 mg/kg/d for 14 d (n = 20)		−0.49 (0.15)
Jarvis et al [16] RCT	South Africa 2007–2010	90	AmB 1 mg/kg/d + 5-FC 100 mg/kg/d (n = 31)	Yes	−0.49 (0.15)
			AmB 1 mg/kg/d + 5-FC 100 mg/kg/d + IFN-γ 100 µg days 1 & 3 (n = 29)		−0.64 (0.27)
			AmB 1 mg/kg/d + 5-FC 100 mg/kg/d + IFN-γ 100 µg days 1, 3, 5, 8, 10, & 12 (n = 30) (AmB + 5-FC for 14 d in all arms)		−0.64 (0.22)

Abbreviations: 5-FC, 5-fluorocytosine; AmB, amphotericin B; ART, antiretroviral therapy; CFU, colony-forming units; EFA, early fungicidal activity; Fluc, fluconazole; IFN, interferon; RCT, randomized controlled trial; SD, standard deviation; Vori, voriconazole.

^a^ The 9 studies were conducted in 5 sites: Sappasitprasong Hospital, Ubon Ratchathani, Thailand; GF Jooste Hospital, Cape Town, and Edendale Hospital, Pietermaritzburg, South Africa; Kamuzu Central Hospital/University of North Carolina Project, Lilongwe, Malawi; and Mbarara University Hospital, Uganda. Exclusion criteria at all clinical trials were an alanine aminotransferase level >5 times the upper limit of normal (>200 IU/mL), neutrophil count <500 × 10^6^ cells/L, platelet count <50 000 × 10^6^ cells/L, pregnancy, lactation, previous serious reaction to study drugs, or concomitant medication contraindicated with study drugs (cisapride and the class of antihistamines including terfenadine and astemizole). Eight hundred ninety-six patients were screened for inclusion in the clinical trials; 523 met eligibility criteria. Reasons for exclusion were ART use in 162, inability to obtain consent in 65 (usually due to reduced Glasgow Coma Score), prior cryptococcal meningitis in 36, patient refusal in 18, death prior to consent in 12, and other reasons including prior antifungal use and study exclusion criteria in 80.

^b^ AmB was administered by intravenous infusion in all studies, and fluconazole by the oral or nasogastric route. Following 2 weeks of induction therapy, patients received 8 weeks of oral fluconazole consolidation therapy (400–800 mg/d), then maintenance therapy (fluconazole 200 mg/d).

### Participants and Procedures

Following informed consent, HIV-positive adults with CM, diagnosed by cerebrospinal fluid (CSF) India ink or cryptococcal antigen testing (Meridian Cryptococcal Latex Agglutination System, Meridian Bioscience) were enrolled consecutively. All the trials had similar inclusion/exclusion criteria (Table 1). Patients already receiving antiretroviral therapy (ART) and those with previous episodes of CM were excluded from this analysis. Induction therapy differed by study as described previously [5, 9–16]. Following 2 weeks' induction therapy, patients received 8 weeks of oral fluconazole consolidation (400–800 mg/day) then maintenance therapy (fluconazole 200 mg/day). Routine drug level monitoring was not performed. ART was started 2–6 weeks after starting antifungal therapy in accordance with local protocols (ART was not routinely available during the earliest trial [9]). Cohorts in Thailand, Uganda, and Malawi were followed for 10 weeks, and those in South Africa for 12 months.

### Evaluations and Outcomes

On study enrollment, detailed history and clinical examination findings were recorded. Lumbar punctures with OP measurements and quantitative CSF cultures were performed on days 1, 3, 7, and 14. Patients with a markedly elevated OP (>30 cm) or symptoms of raised intracranial pressure had more frequent lumbar punctures [18]. CSF cell count, protein, and glucose levels were determined. CSF interferon gamma (IFN-γ), tumor necrosis factor alpha (TNF-α), and interleukin 6 (IL-6) concentrations were measured in patients from the Thai and South African sites using the Luminex multianalyte platform and Bio-Rad cytokine kits [19]. Cryptococcal clearance was calculated as the decrease in log colony-forming units (CFU) per milliliter of CSF per day derived from the slope of the linear regression of log CFU per milliliter against time for each patient [9]. Baseline blood tests included hematology, renal and liver function, CD4 cell counts, and, where available, plasma HIV load. The primary outcome in all studies was rate of decrease in CSF cryptococcal CFU (ie, EFA). Secondary outcomes included mortality at 2 and 10 weeks. Cryptococcal meningitis IRIS (CM-IRIS) was diagnosed according to uniform criteria [20]. In patients who died, the presumed cause of death was ascertained by 2 study clinicians.

### Statistical Analysis

Data were analyzed using Stata software, version 11 (StataCorp). Variables were compared using Kruskal-Wallis, χ^2^, χ^2^ for trend, Fisher exact, or *t* tests. Relationships between continuous variables were examined using the Pearson correlation coefficient or Spearman log-rank test. Multivariable logistic regression models were constructed using stepwise regression with the primary objective of determining the clinical and microbiological factors at baseline associated independently with all-cause mortality (as measured at 2 and 10 weeks). A predictive modeling strategy was used in which variables were selected for model inclusion based upon (1) a priori knowledge from previous studies (CD4 cell count), and (2) association with outcome in univariable analysis. Variables associated with mortality in univariable analysis (*P* ≤ .1) were included in the first fit of the multivariable model and retained, based on likelihood ratio testing, if they significantly improved model fit, to obtain the most parsimonious model identifying predictors of mortality. Clustering by individual study was accounted for using a hierarchical mixed effects model including a random-effects term for “study.” An a priori decision was made to adjust the multivariable model for amphotericin (AmB) vs fluconazole-based treatment as a potential confounder in the relationship between baseline factors and outcomes. Exploring the effect of treatment on outcome, after adjusting for other predictors, was a secondary objective. Patients with missing outcome data were censored from the main analysis, with sensitivity analyses performed assuming that all patients lost were either dead or alive. Further models were constructed to examine the baseline factors associated with altered mental status, baseline fungal burdens, and CSF opening pressure; to examine the impact of ART timing and IRIS on longer-term outcomes; and to describe the relationship between EFA and outcome. EFA was modeled both as a single linear term for each patient as previously described [17] and as a time-updated variable in a Cox regression. In the group with 1 year of follow-up data, Kaplan-Meier survival curves were compared using the Mantel-Haenszel log-rank test.

## RESULTS

### Baseline Characteristics and Outcome

After screening 896 patients, 523 met eligibility criteria for inclusion in the clinical trials, consented to participation, and were included. Of these, we studied the 501 ART-naive patients with a first episode of CM (Tables 1 and 2). The median age was 34 years, and 52% were male. All had confirmed HIV infection; 76% were known to be HIV positive at time of presentation, diagnosed a median of 152 days (interquartile range [IQR], 44–745 days) earlier; the remainder tested HIV positive at study enrollment. Male patients presented with a longer median reported duration of symptoms than female patients (14 vs 10 days; *P* = .004). The median CD4 count was 23 cells/µL. Amphotericin B deoxycholate ([AmB] 0.7–1 mg/kg/day) induction treatment was used in 80% of patients, and 20% received fluconazole-based induction (median, 1200 mg/day) without AmB. All-cause mortality was 17% at 2 weeks and 34% at 10 weeks (Tables 3 and 4). Of patients in care at 2 weeks (n = 410), 244 were started on ART a median of 30 days (IQR, 26–42 days) after starting antifungal therapy. Nine patients were lost to follow-up at 2 weeks, and 17 at 10 weeks.

**Table 2.** Baseline Characteristics of the Cohort

Characteristic	Variable	No.	% (No.) or Median (IQR)
Demographics	Age, y	499	34 (29–39)
	Sex, male	501	52% (260)
History	Concurrent tuberculosis	419	25% (123)
	Duration of symptoms, d	458	14 (7–21)
Symptoms	Headache	496	99% (489)
	Febrile symptoms	497	57% (280)
	Visual symptoms	493	51% (250)
	Hearing loss	415	14% (60)
	Seizures	496	19% (94)
	Nausea/vomiting	494	54% (266)
	Cough	494	35% (173)
Signs	Fever, >37.5°C	479	23% (112)
	Tachycardia, >100 bpm	491	19% (91)
	Hypotension, <90/50 mm Hg	485	3% (15)
	Tachypnea, >20 bpm	463	19% (89)
	Altered mental status	499	25% (123)
	Meningism	492	75% (369)
	Papilledema	311	12% (36)
	Decreased visual acuity, <6/6	361	39% (141)
	Cranial nerve lesion	434	13% (57)
Investigations	Raised OP >25 cm CSF	450	51% (230)
	Raised OP >30 cm CSF	450	38% (173)
	CSF white cell count, ×10^6^/L	461	15 (1–57)
	CSF protein, g/dL	392	0.7 (0.4–1.3)
	CSF glucose, mg/dL	374	39.6 (25.2–50.5)
	CSF CRAG, titer^a^	247	1:1024 (1:512–4096)
	QCC, log_10_ CFU/mL^a^	496	5.30 (4.5–5.9)
	CD4, cells/µL	456	24 (10–50)
	Log_10_ VL, copies/mL	368	5.15 (4.7–5.5)
Outcomes	2-week mortality	492	17% (82)
	10-week mortality	484	34% (163)
	Time admission to death, d	161	13 (5–310)

Abbreviations: CFU, colony-forming units; CRAG, cryptococcal antigen; CSF, cerebrospinal fluid; IQR, interquartile range; OP, opening pressure; QCC, quantitative cryptococcal culture; VL, HIV load.

^a^ See Supplementary Figure 1 for a description of the relationship between CSF CRAG and QCC.

**Table 3.** Associations Between Baseline Variables and 2-Week Mortality

Variable	Category	No.	2-wk Mortality	OR (95% CI), Univariable	*P* Value	AOR (95% CI), Multivariable^a,b^	*P* Value
Age	<50 y	462	16% (73)	1	.009	1	.02
	≥50 y	24	38% (9)	3.2 (1.3–7.8)		3.9 (1.4–11.1)	
Sex	Female	237	15% (36)	1	.5		
	Male	255	18% (46)	1.2 (.7–2.0)			
Seizures	No	397	14% (56)	1	.007		
	Yes	91	27% (25)	2.2 (1.2–3.9)			
Mental status	Normal	372	11% (39)	1	<.001	1	<.001
	Abnormal	119	36% (43)	4.8 (2.9–8.0)		3.1 (1.7–5.9)	
Weight	<50 kg	175	17% (30)	1	.13		
	≥50 kg	282	10% (30)	0.7 (.4–1.1)			
Pulse	≤100 bpm	395	15% (58)	1	.033		
	>100 bpm	88	24% (21)	1.9 (1.1–3.3)			
Respiratory rate	≤20 bpm	368	13% (49)	1	.002		
	>20 bpm	87	26% (23)	2.6 (1.4–4.7)			
CD4 cell count	<25 cells/µL	229	17% (39)	1	.05	1	.07
	25–49 cells/µL	106	8% (8)	0.4 (.2–.9)		0.4 (.2–.9)	
	50–99 cells/µL	74	7% (5)	0.4 (.1–.9)		0.5 (.2–1.4)	
	≥100 cells/µL	39	13% (5)	0.7 (.3–1.9)		1.1 (.4–3.2)	
Hemoglobin	≥7.5 g/dL	429	15% (65)	1	.02		
	<7.5 g/dL	28	32% (9)	2.8 (1.2–6.7)			
White blood cell count	≤10 × 10^9^/L	382	14% (53)	1	<.001	1	.002
	>10 × 10^9^/L	21	48% (10)	6.7 (2.6–17.7)		8.7 (2.5–30.2)	
CSF opening pressure	≤25 cm CSF	216	18% (38)	1	.488		
	>25 cm CSF	226	16% (37)	0.8 (.5–1.4)			
CSF white cell count	≤20 × 10^6^/L	272	20% (54)	1	.017		
	>20 × 10^6^/L	183	11% (20)	0.5 (.3–0.9)			
QCC	1st tertile	163	9% (15)	1	<.001	1.4 (1.0–1.8)	.02
	2nd tertile	162	14% (22)	1.5 (.8–3.1)		(per log_10_ CFU/mL increase)^c^	
	3rd tertile	163	27% (44)	3.6 (1.9–6.8)			
Treatment	Fluconazole	99	26% (26)	1	.005	1	.05
	Amphotericin	393	14% (56)	0.5 (.3–.8)		0.5 (.3–1.0)	

Abbreviations: AOR, adjusted odds ratio; CI, confidence interval; CFU, colony-forming units; CSF, cerebrospinal fluid; OR, odds ratio; QCC, quantitative cryptococcal culture.

ORs and 95% CIs for both univariable and multivariable associations are adjusted for clustering by study using a random-effects term for “study” in a hierarchical mixed-effects logistic regression model. There was very little evidence for significant clustering by study in either the 2-week or 10-week model (likelihood-ratio test of ρ = 0, *P* = .498 at both 2 and 10 weeks).

Numbers of patients included in each analysis are indicated in the table. A complete records analysis was performed rather than multiple imputation as there were relatively few missing data points in the key exposure and outcome variables, and missing variables in important exposure variables such as CD4 cell count were thought to be missing not at random, meaning imputation would not provide less biased results. It was suspected that lower values were associated with more advanced disease and that blood tests were deferred in the sickest patients until they could consent to CD4 testing, meaning patients with the lowest values may have been less likely to have a baseline test. A sensitivity analysis in which all patients lost to follow-up were assumed to be either alive or dead did not alter the findings of either the 2 or 10-week model.

^a^ Only variables included in the multivariable model are shown. Adjusted for treatment, CD4 count, age, mental status, and fungal burden (see footnote b). Number included in final model = 445.

^b^ Peripheral white cell count was significantly associated with 2-week mortality after adjustment, but was not included in the final model as observations were missing for 90 patients. Its inclusion in a model considering only the patients with complete data (n = 370) did not alter the magnitude or significance of the associations seen in the full model.

^c^ QCC is shown in the univariable analysis as a categorical variable for ease of interpretation, but was included in the multivariable model as a continuous variable to give a better fit.

**Table 4.** Associations Between Baseline Variables and 10-Week Mortality

Variable	Category	No.	10-wk Mortality	OR (95% CI), Univariable	*P* Value	AOR (95% CI), Multivariable^a,b^	*P* Value
Age	<50 y	454	33% (148)	1	.014	1	.009
	≥50 y	24	58% (14)	2.9 (1.2–6.8)		4.0 (1.4–11.4)	
Sex	Female	231	34% (79)	1	.815		
	Male	250	34% (84)	1.0 (.6–1.4)			
Seizures	No	389	33% (127)	1	.912		
	Yes	91	37% (34)	1.0 (.6–1.6)			
Mental status	Normal	366	25% (90)	1	<.001	1	<.001
	Abnormal	117	62% (73)	5.2 (3.3–8.3)		2.8 (1.6–4.7)	
Weight	<50 kg	173	39% (68)	1	.003	1	.004
	≥50 kg	277	25% (68)	0.5 (.3–.8)		0.6 (.4–1.0)	
Pulse	≤100 bpm	389	31% (121)	1	.010		
	>100 bpm	86	45% (39)	1.9 (1.2–3.1)			
Respiratory rate	≤20 bpm	363	30% (110)	1	.006		
	>20 bpm	84	45% (38)	2.0 (1.2–3.4)			
CD4 cell count	<25 cells/µL	226	35% (80)	1	.03^c^	1	.781
	25–49 cells/µL	102	30% (30)	0.7 (.4–1.2)		0.8 (.5–1.4)	
	50–99 cells/µL	73	23% (17)	0.6 (.3–1.0)		0.8 (.4–1.5)	
	≥100 cells/µL	39	23% (9)	0.5 (.2–1.2)		0.7 (.3–1.9)	
Hemoglobin	≥7.5 g/dL	423	31% (133)	1	.008	1	.02
	<7.5 g/dL	27	56% (15)	3.0 (1.3–6.4)		3.0 (1.2–7.4)	
White blood cell count	≤10 × 10^9^/L	377	30% (114)	1	.001	1	.02
	>10 × 10^9^/L	21	63% (13)	4.7 (1.8–12.2)		4.0 (1.3–12.6)	
CSF opening pressure	≤25 cm CSF	213	39% (83)	1	.009	1	.002
	>25 cm CSF	223	30% (66)	0.6 (.4–.9)		0.4 (.3–.7)	
CSF white cell count	≤20 × 10^6^/L	268	35% (93)	1	.461		
	>20 × 10^6^/L	179	31% (55)	0.9 (.6–1.3)			
QCC	1st tertile	161	24% (38)	1	<.001	1.3 (1.1–1.7)	.007
	2nd tertile	161	32% (52)	1.5 (.9–2.4)		(per log_10_ CFU/mL increase)^d^	
	3rd tertile	158	46% (72)	2.8 (1.7–4.5)			
Treatment	Fluconazole	99	53% (52)	1	<.001	1	.02
	Amphotericin	385	29% (111)	0.4 (.2–.6)		0.5 (.3–.9)	

Abbreviations: AOR, adjusted odds ratio; CI, confidence interval; CFU, colony-forming units; CSF, cerebrospinal fluid; OR, odds ratio; QCC, quantitative cryptococcal culture.

ORs and 95% CIs for both univariable and multivariable associations are adjusted for clustering by study using a random-effects term for “study” in a hierarchical mixed-effects logistic regression model. There was very little evidence for significant clustering by study in either the 2-week or 10-week model (likelihood-ratio test of ρ = 0, *P* = .498 at both 2 and 10 weeks).

Numbers of patients included in each analysis are indicated in the table. A complete records analysis was performed rather than multiple imputation as there were relatively few missing data points in the key exposure and outcome variables, and missing variables in important exposure variables such as CD4 cell count were thought to be missing not at random, meaning imputation would not provide less biased results. It was suspected that lower values were associated with more advanced disease, and that blood tests were deferred in the sickest patients until they could consent to CD4 testing, meaning patients with the lowest values may have been less likely to have a baseline test. A sensitivity analysis in which all patients lost to follow-up were assumed to be either alive or dead did not alter the findings of either the 2 or 10-week model.

^a^ Only variables included in the multivariable model are shown. Adjusted for treatment, CD4 count, age, mental status, weight and fungal burden (see footnote b). Number included in final model = 413.

^b^ Peripheral white cell count, anemia, and raised CSF opening pressure were significantly associated with 10-week mortality after adjustment, but not included in the final model to prevent missing observations, markedly limiting the size of the model. Inclusion in a model considering only the patients with complete data (n = 391 for hemoglobin, n = 343 for peripheral white count, and n = 374 for raised CSF opening pressure) did not alter the magnitude or significance of the associations seen in the full model.

^c^ Test for trend.

^d^ QCC is shown in the univariable analysis as a categorical variable for ease of interpretation, but was included in the multivariable model as a continuous variable to give a better fit.

### Independent Associations With Outcome

Four variables independently predicted mortality at 2 weeks in multivariable analysis: altered mental status, high baseline CSF fungal burden (measured by either quantitative cryptococcal culture [QCC] or cryptococcal antigen titer, which were closely correlated; Supplementary Figure 1), older age, and high peripheral white blood cell count (Table 3). Patients with altered mental status at presentation had a 3-fold increase in mortality at 2 weeks; there was an incremental 1.4 odds increase in mortality with each log_10_ CFU/mL increase in fungal burden; and patients aged ≥50 years were almost 4 times more likely to die than those aged <50 years.

Seven baseline parameters were associated independently with 10-week mortality: altered mental status, high baseline fungal burden, older age, low body weight, low CSF opening pressure (OP ≤25 cm CSF), high peripheral blood white cell count, and anemia (Table 4). AmB treatment was associated independently with lower mortality at both 2 and 10 weeks; however it should be noted that fluconazole-treated patients were predominantly from the lowest-resource settings (97% Uganda or Malawi).

### Altered Mental Status

At presentation, 25% of patients had altered mental status. In a multivariable regression model including 409 patients with complete data, the strongest predictor of altered mental status was male sex (adjusted odds ratio [AOR], 2.2; 95% confidence interval [CI], 1.3–3.7; *P* = .003). Additional variables associated independently with altered mental status were age >50 years (AOR, 1.4; 95% CI, 1.1–2.0; *P* = .02) and very high CSF opening pressure (>30 cm CSF; AOR, 1.8; 95% CI, 1.1–3.0; *P* = .02). Altered mental status was not associated with any other variables examined including baseline fungal burden, CD4 count, or CSF white cell count in adjusted analyses.

### Baseline CSF Fungal Burden

**Figure 1.**
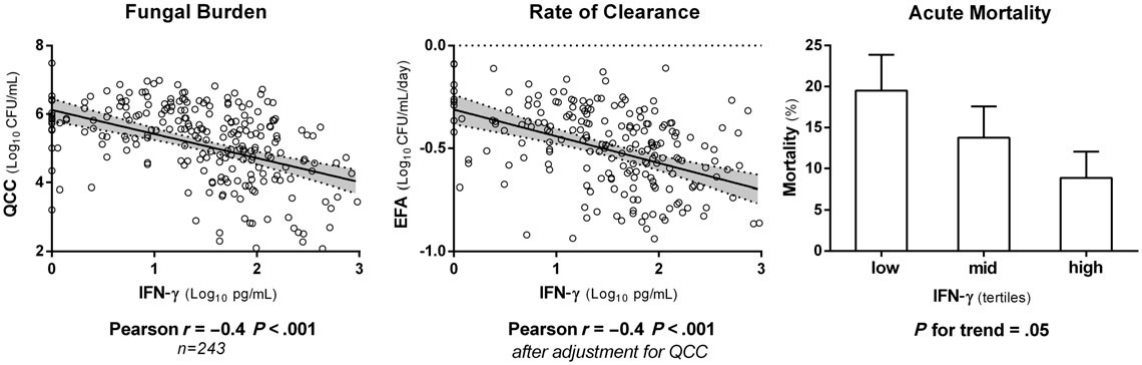
Associations between cerebrospinal fluid (CSF) interferon gamma (IFN-γ) concentrations and baseline fungal burden, rate of clearance of infection (EFA), and 2-week mortality in the 243 Thai and South African patients with CSF cytokine measurements. CSF IFN-γ concentration was strongly associated with rate of clearance of infection, with a 0.10 log_10_ CFU/mL/day (95% confidence interval, .06–.15) increase in EFA for every log_10_ picogram increment in IFN-γ concentration. Abbreviations: CFU, colony-forming units; EFA, early fungicidal activity; IFN, interferon; QCC, quantitative cryptococcal culture.

CSF QCCs were negatively correlated with CD4 count, CSF white cell count, CSF protein, and CSF proinflammatory cytokines (IL-6, IFN-γ, and TNF-α). The strongest correlation was with CSF IFN-γ (Pearson *r* = −0.4, *P* < .001; Figure 1). There was no significant correlation between QCC and CSF OP (Table 5).

**Table 5.** Correlations Between Baseline Cerebrospinal Fluid Fungal Burden, Derived From Quantitative Cryptococcal Cultures^a^

Correlation With QCC^b^	No.	Correlation Coefficient (*r*)	95% CI	*P* Value
CD4 cell count (cells/µL)	452	−0.24	−.33 to −.15	<.001
CSF opening pressure (cm CSF)	446	0.05	−.05 to .14	.330
CSF white cell count (×10^6^/L)	458	−0.30	−.39 to −.21	<.001
CSF protein (g/dL)	389	−0.29	−.38 to −.19	<.001
CSF TNF-α (log_10_ pg/mL)	242	−0.20	−.32 to −.07	.002
CSF IL-6 (log_10_ pg/mL)	241	−0.15	−.27 to −.02	.024
CSF IFN-γ (log_10_ pg/mL)	243	−0.40	−.50 to −.29	<.001

Abbreviations: CI, confidence interval; CSF, cerebrospinal fluid; IFN, interferon; IL-6, interleukin 6; QCC, quantitative cryptococcal culture; TNF, tumor necrosis factor.

^a^ Log_10_ colony-forming units per milliliter CSF.

^b^Correlations were assessed using Spearman log-rank test for CD4 count, CSF opening pressure, CSF white cell count, and CSF protein; and Pearson correlation coefficient for TNF-α, IL-6, and IFN-γ, which were approximately normally distributed when log transformed.

### CSF Opening Pressure

Raised baseline CSF OP (>25 cm CSF) was present in 51% of the cohort (n = 230). Raised pressure was associated with papilledema (OR, 2.6; 95% CI, 1.1–5.8; *P* = .02); however, other than the association between very high CSF opening pressures (OP >30 cm) and mental status described above, there were no other significant associations between high OP and clinical variables. Raised OP correlated with increasing CSF TNF-α concentrations (Spearman *r* = 0.2, *P* = .008), but not with IFN-γ or IL-6. Although there was no significant correlation between QCC and baseline CSF OP, high baseline QCC was necessary but insufficient for development of a high day 1 and day 14 OP (Supplementary Figure 2).

### Early Fungicidal Activity

EFA was associated with outcome, as shown previously among a subset of 262 patients [17]. A slope measurement was available in 450 of the 501 patients, and in 129 of the 163 patients who died. Mean EFA of those who died at 2 weeks was −0.24 (SD, 0.25) log_10_ CFU/mL/day vs −0.42 (SD, 0.25) log_10_ CFU/mL/day in survivors (*P* < .001). In those who died at 10 weeks, EFA was −0.34 (SD, 0.27) log_10_ CFU/mL/day vs −0.43 (SD, 0.24) log_10_ CFU/mL/day in survivors (*P* < .001). EFA remained independently associated with mortality after adjusting for altered mental status and fungal burden (mean difference in EFA between survivors and fatal cases at 2 weeks, −0.15 log_10_ CFU/mL/day; 95% CI, .07−.22; *P* < .001). When the serial counts were fitted as a time-dependent variable, the adjusted hazard ratio for death within the first 2 weeks was 1.8 (95% CI, 1.2–2.5; *P* = .002) for each unit increase in the log_10_ CFU count. The mean EFA was greater for amphotericin-based compared with fluconazole-based induction treatment (difference, 0.32 log_10_ CFU/day; 95% CI, .27–.36; *P* < .001). EFA was associated independently with amphotericin-based treatment, baseline organism count, and CSF IFN-γ level (Figure 1).

### Long-term Outcomes, ART Timing, and IRIS

**Figure 2.**
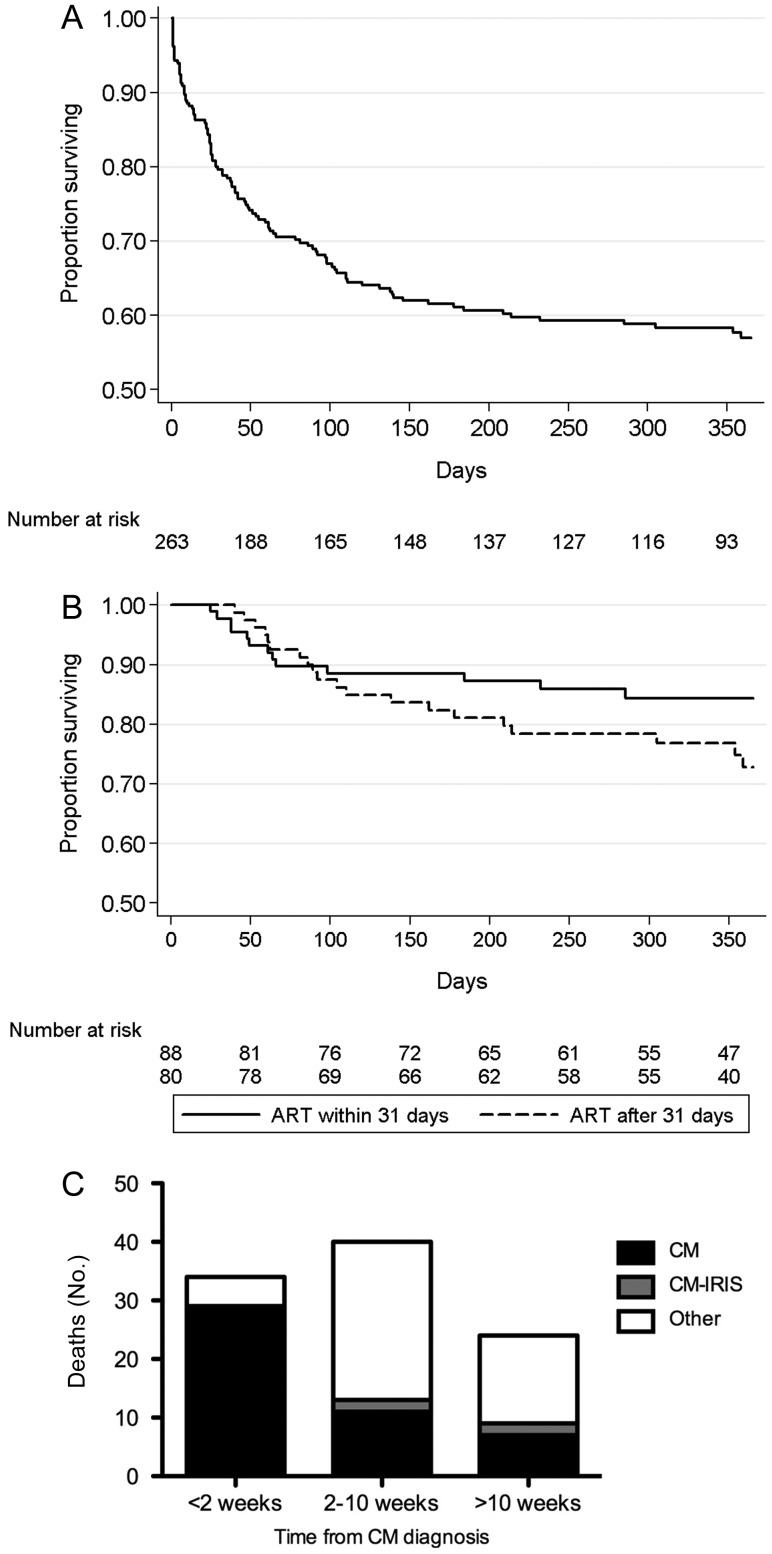
Kaplan-Meier survival curves. *A*, Survival in the 263 South African amphotericin B (AmB)–treated patients followed up for 1 year. *B*, Survival from enrollment in the subset of patients who started antiretroviral therapy (ART), stratified by ART timing (before and after the median of 31 days; *P* = .15). Only patients surviving to ART initiation are shown. *C*, Cause of death data (as determined by study clinicians) in the cohort of 263 South African AmB-treated patients followed up for 1 year, split by time from diagnosis. “Other” included tuberculosis (n = 11), bacterial sepsis (n = 8), bacterial pneumonia (n = 7), nonspecified infections (n = 9), Kaposi sarcoma (n = 2), lymphoma (n = 1), non–cryptococcal meningitis immune reconstitution inflammatory syndrome (n = 3), ART toxicity (n = 2), diarrhea/wasting syndrome (n = 4), and decompensation of chronic liver disease (n = 1). Abbreviations: ART, antiretroviral therapy; CM, cryptococcal meningitis; IRIS, immune reconstitution inflammatory syndrome.

Among the 266 patients enrolled in South Africa, survival analysis was restricted to the 263 patients treated with AmB-based regimens. The median age was 33 years (IQR, 29–39 years), 42% of patients (n = 110) were male, median CD4 count was 28 cells/µL (IQR, 12–57 cells/µL), and 15% had altered mental status. Of 179 patients surviving to 10 weeks and in care, median follow-up was 352 days (IQR, 209–409 days). Mortality was 13% at 2 weeks, 30% at 10 weeks, and 41% at the end of follow-up (Figure 2). Of patients surviving to 2 weeks, 85% (n = 171) started ART a median of 31 days (IQR, 23–46 days) after antifungal therapy. IRIS developed in 13% (n = 22) of patients a median of 29 days (IQR, 23–45 days) after starting ART, of whom 18% (n = 4) died. IRIS was associated with day 14 CSF fungal burden (*P* = .007) but not with time to ART (*P* = .4).

Presumed causes (as ascertained clinically by study physicians) were recorded for 91% (98/108) of deaths (Figure 2). Deaths during the initial 2 weeks were primarily attributed to CM (85% [29/34]). Later deaths were mostly attributed to other HIV-related infections or complications (67% [43/64]). Survival in patients who started ART was not associated with IRIS (*P* = .3) or time to ART (*P* = .3) (Figure 2*B*).

## DISCUSSION

This is the largest study examining factors determining outcome in HIV-associated CM. The results emphasize the high acute mortality in patients with CM, even among patients primarily treated with amphotericin-based therapy in research settings. Such patients are likely to have better outcomes than those managed in routine care; hence, these results provide a minimum mortality estimate. One-third of patients had died by 10 weeks after presentation. However, the median time to death of 13 days suggests that improved early interventions could prevent some of these fatalities. Acute deaths were attributed mainly to CM. High fungal burden and slow clearance of infection on treatment, together with altered mental status, were the most important drivers of acute cryptococcal-related mortality. After 2 weeks, other HIV-related causes of death predominated, and the risk factors for mortality in addition to high fungal burden, slow clearance of infection, and altered mental status, included older age, low CD4^+^ cell count, low weight, and anemia, which have been identified previously as predictors of mortality in HIV cohorts in general [21].

High baseline CSF fungal burden was one of the strongest risk factors for mortality. It was associated with a low peripheral CD4 count, reflecting the importance of cell-mediated immunity in controlling cryptococcal infection [22], and with a poor inflammatory response at the site of infection, as evidenced by low CSF white cell counts and low levels of IFN-γ, TNF-α, and IL-6. The rate of fungal clearance was independently related to outcome, with slower clearance associated with higher mortality. This strongly supports the use of EFA as a clinically relevant pharmacodynamic endpoint for phase 2 clinical studies [17]. Higher levels of proinflammatory CSF IFNγ were associated with more rapid rates of fungal clearance during treatment [17, 19] and lower fungal burdens at presentation, demonstrating the importance of IFN-γ in the protective immune response, and reinforcing the rationale for augmentation of proinflammatory responses with IFN-γ immunotherapy as a therapeutic approach [16].

Amphotericin-based treatments were more rapidly fungicidal than fluconazole-based treatments, and the mortality in fluconazole-treated patients was almost double that in amphotericin B-treated patients at 10 weeks. However the majority of fluconazole-treated patients were from the lowest-resource settings, and it is possible that the association between fluconazole use and mortality is confounded by factors relating to study site; however, the association remained statistically robust after adjustment for the other key variables associated with outcome, including abnormal mental status and baseline fungal burden. The clear association between slow rates of fungal clearance and poor outcomes provides a strong argument in favor of rapidly fungicidal initial treatments, and more widespread use of rapidly fungicidal amphotericin B combination therapy is likely to reduce early mortality.

The pathophysiological basis of altered mental status, the other main risk factor for mortality, remains unclear. The independent associations of altered mental status with male sex and older age have not been reported previously. High CSF opening pressure (>30 cm) was associated independently with altered mental status, but was not contributory to altered mental status in the majority of cases; half of those with altered mental status did not have markedly raised pressures. Of note, altered mental status was not associated with CD4 count, CSF white cell count, or fungal burden.

High CSF opening pressure was not associated with increased mortality in this cohort, in contrast to earlier reports [23]. This may have been a result of management: all patients routinely had 4 lumbar punctures over the first 2 weeks of treatment, and raised pressures were managed according to established guidelines [18]. A novel finding of this analysis was that raised CSF opening pressures at baseline, in patients managed according to these guidelines, were associated with improved outcomes at 10 weeks. It is possible that proinflammatory CSF cytokine responses (TNF-α was associated with raised pressure) may be protective in situations where raised OP is appropriately managed, or that large volume CSF drainage is beneficial over and above its role in reducing pressure [23]. These findings emphasize the importance of CSF pressure management in patients with CM, and highlight the need for widened access to manometers to manage pressure safely in centers in Africa with the highest burden of disease.

Long-term survival in the cohort of South African patients with access to AmB and ART was good, provided patients survived the acute period. ART was usually started between 3 and 6 weeks after antifungal therapy. Within this time frame, there was no association between earlier ART initiation and the development of subsequent IRIS. Patients who developed IRIS did not have higher overall mortality. The majority of deaths after 2 weeks were attributed to other HIV-related illnesses that may have been preventable through earlier initiation of ART. In the context of amphotericin induction, ART initiation nearer to 3 rather than 6 weeks after starting antifungal therapy may prevent some of the later HIV-related mortality, while not substantially increasing the risk of IRIS.

A potential limitation of this analysis, derived from multiple cohorts, is possible residual confounding due to unmeasured study specific effects, relating to temporal or geographic differences between studies. However a key strength of this cohort is the extensive prospectively collected baseline data, allowing adjustment to minimize confounding. There was little evidence of clustering by study within the hierarchical model, and the robustness of the key conclusions was further supported by consistency across univariable and multivariable analyses, and the sensitivity analyses performed. Levels of missing data among outcomes and the key predictor variables were low, reducing the risk of bias.

In summary, these data provide a rationale for several strategies to improve outcomes. First, earlier diagnosis of CM should be possible, resulting in lower fungal loads at presentation and a reduction in mortality. Clinicians should have a low threshold for lumbar puncture in HIV-positive patients presenting with headache. Novel point-of-care antigen tests [24, 25] should now facilitate earlier diagnosis. Given the high proportion of patients presenting with CM who have already been diagnosed with HIV (76%), screening for subclinical infection with point-of-care antigen tests and preemptive antifungal treatment, along with early ART initiation, could prevent a substantial proportion of clinical disease from developing [26–28]. Second, increasing access to the most fungicidal AmB-based regimens is a priority in settings with a high incidence of CM [29–31], in particular sub-Saharan Africa. Last, prompt initiation of ART is required to address the substantial proportion of deaths in these patients that are HIV but not CM related.

## Supplementary Data

Supplementary materials are available at *Clinical Infectious Diseases* online (http://cid.oxfordjournals.org/). Supplementary materials consist of data provided by the author that are published to benefit the reader. The posted materials are not copyedited. The contents of all supplementary data are the sole responsibility of the authors. Questions or messages regarding errors should be addressed to the author.

Supplementary Data
