# Supplementary material for: Determinants of Mortality in a Combined Cohort of 501 Patients With HIV-Associated Cryptococcal Meningitis: Implications for Improving Outcomes
Source: Clin Infect Dis. 2013 Dec 6;58(5):736–45. doi: 10.1093/cid/cit794 (PMC3922213; doi:10.1093/cid/cit794)
Supplement: Supplementary Data [file supp_58_5_736__index.html]

Determinants of Mortality in a Combined Cohort of 501 Patients With HIV-Associated Cryptococcal Meningitis: Implications for Improving Outcomes — Determinants of Mortality in a Combined Cohort of 501 Patients With HIV-Associated Cryptococcal Meningitis: Implications for Improving Outcomes — Supplementary Data 

# Determinants of Mortality in a Combined Cohort of 501 Patients With HIV-Associated Cryptococcal Meningitis: Implications for Improving Outcomes

## Supplementary Data

Supplementary Data

**Files in this Data Supplement:**

- Supplementary Data - Pdf file
