# Supplementary material for: Determinants of Mortality in a Combined Cohort of 501 Patients With HIV-Associated Cryptococcal Meningitis: Implications for Improving Outcomes
Source: Clin Infect Dis. 2013 Dec 6;58(5):736–45. doi: 10.1093/cid/cit794 (PMC3922213; doi:10.1093/cid/cit794)

Supplementary Figure s1. Relationship between baseline CSF cryptococcal antigen (CRAG) titres and fungal burden determined by quantitative cryptococcal culture (QCC).

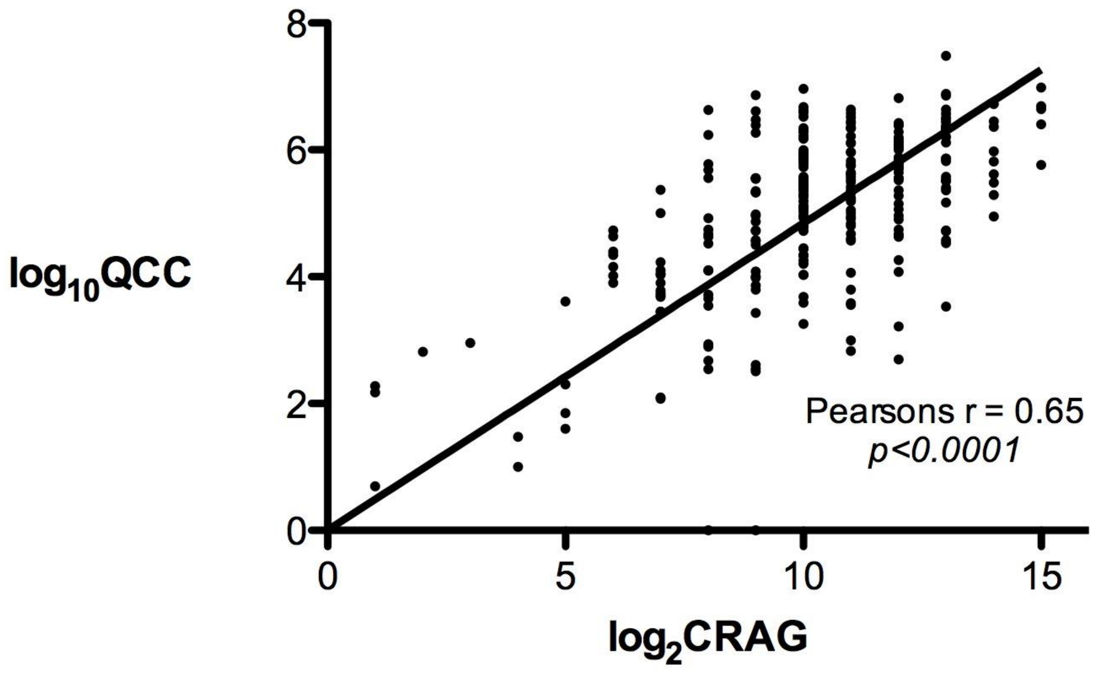

Supplementary Figure s2. Relationship between baseline CSF opening pressure and baseline fungal burden in a cohort of 501 patients with HIV-associated cryptococcal meningitis (Spearman's r=0.05, p=0.3).

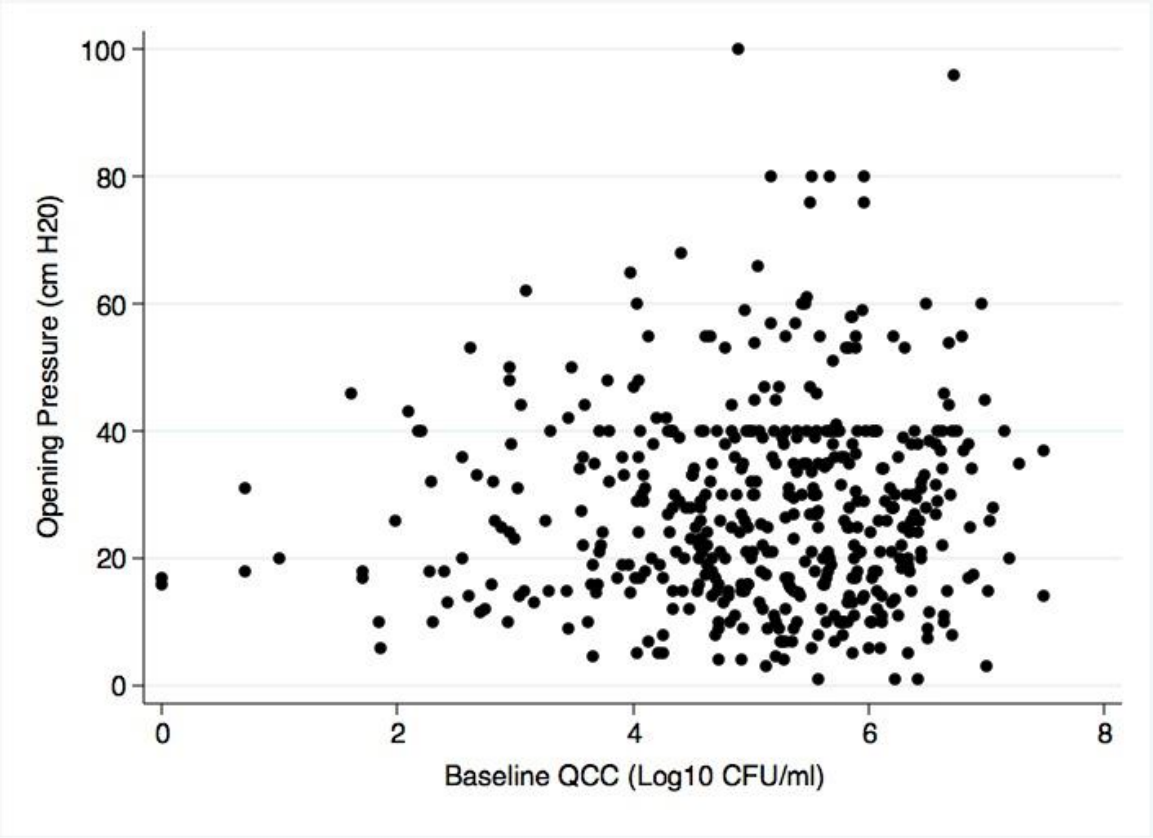

Supplement: Supplementary Data [file supp_cit794_cit794supp.pdf]
